# Clinical Application of Extracorporeal Membrane Oxygenation in the Treatment of Fulminant Myocarditis

**DOI:** 10.31083/j.rcm2504114

**Published:** 2024-03-26

**Authors:** Zhijun Fan, Junlin Wen, Binfei Li, Xiaozu Liao

**Affiliations:** ^1^The First Clinical Medical College, Guangdong Medical University, 524002 Zhanjiang, Guangdong, China; ^2^Department of Anesthesiology, Zhongshan City People’s Hospital, 528403 Zhongshan, Guangdong, China

**Keywords:** extracorporeal membrane oxygenation, mechanical circulatory support, fulminant myocarditis, myocarditis, cardiogenic shock

## Abstract

Fulminant myocarditis (FM) is a rare but serious clinical syndrome which can be characterized by the rapid deterioration of cardiac function, with cardiogenic shock (CS) and arrhythmic electrical storms being common presentations, often requiring adjunctive support with mechanical circulatory devices. With the development of mechanical circulatory support (MCS) devices, there are now more and more studies investigating the application of MCS in FM patients, and the use of extracorporeal membrane oxygenation (ECMO) to treat FM has shown good survival rates. This review elucidates the treatment of FM, and the application and clinical outcomes associated with ECMO intervention.

## 1. Introduction

Myocarditis, an inflammatory lesion of the myocardium, is induced by various infectious or non-infectious factors and is generally classified into non-fulminant myocarditis (NFM) and fulminant myocarditis (FM). FM constitutes a distinct clinical subtype of myocarditis, characterized by abrupt, severe, and widespread cardiac inflammatory damage. It features rapid onset and swift progression, leading to early refractory hemodynamic instability and severe circulatory failure, often accompanied by multi-organ failure, posing a significant life-threatening risk to the patient [1, 2]. In cases where there is no improvement after conventional supportive therapy with medications, temporary mechanical circulatory devices such as extracorporeal membrane oxygenation (ECMO) are often needed to support the patient through the acute phase. This review provides an overview of the definition, etiology, epidemiology, and diagnosis of FM, and focuses on the treatment of FM, the clinical outcomes of ECMO in the treatment of FM, and the advances in its application, as well as discussing some of the clinical issues that need to be addressed, such as the optimal time for ECMO initiation and ECMO-related complications.

### 1.1 Definition

Acute myocarditis (AM) is an inflammatory cardiomyopathy caused by various etiologies, including viral infections, direct injury, or immune responses, and is common in healthy young adults and is more common in men [3]. It presents with reduced cardiac contractile and diastolic function, accompanied by arrhythmias. The period from the onset of symptoms to diagnosis usually does not exceed one month. Individual clinical presentations vary widely, from asymptomatic or mild symptoms to severe cardiac arrest and sudden death [4]. The more common prodromal symptoms include chest pain, fever, dyspnea, and syncope [5]. The severity of myocarditis is largely related to the location and extent of the lesion, and the course is mostly self-limiting. FM is the most clinically severe form of acute myocarditis. It usually occurs within one month of the onset of prodromal symptoms, and requires hemodynamic supportive therapy with medications or mechanical circulatory support (MCS) devices due to severe hemodynamic compromise due to cardiogenic shock (CS), without an ischemic etiology or preexisting cardiomyopathy [6]. Historically, FM is usually diagnosed at autopsy [2].

### 1.2 Etiology and Pathophysiology

The major causes of FM include infections caused by a variety of pathogens (e.g., viruses, bacteria, parasites, Trypanosoma cruzi, etc.), autoimmune diseases (e.g., systemic lupus erythematosus, Chugg-Strauss syndrome, etc.), toxic toxins (e.g., heavy metals, anthracyclines, cocaine, etc.), and adverse drug reactions (e.g., immune checkpoint inhibitors (ICIs), vaccines, etc.) [5, 7]. The initial pathogenesis of FM is similar to that of NFM, with viral infections being the predominant causative factor. Common viruses include Coxsackievirus, adenovirus, cytomegalovirus, EB virus, and influenza virus [5]. These viruses can invade the human host through the respiratory or digestive tract, infiltrate myocardial cells, and extensively replicate, resulting in degeneration, apoptosis, or even necrosis of myocardial cells. This induces direct myocardial damage, and the released cytokines can further harm other tissues and organs, leading to systemic multi-organ damage. Additionally, they can trigger a cytokine storm and incite the generation of autoantibodies against myocardial cells, culminating in severe autoimmune responses [8, 9]. While the majority of AM patients recover spontaneously after viral clearance, some continue to undergo pathological myocardial remodeling due to persistent inflammatory reactions, ultimately progressing to dilated cardiomyopathy or even chronic heart failure [1]. Such patients necessitate heart transplantation (HTx) or implantation of a permanent ventricular assist device for life-sustaining support. Generally, FM can be diagnosed when AM manifests suddenly and advances rapidly, concomitant with severe heart failure, hypotension, or CS, necessitating treatment involving inotropic drugs, vasopressors, or MCS [1].

### 1.3 Epidemiology

The current incidence of myocarditis remains uncertain. Prior to the Corona Virus Disease 2019 (COVID-19) pandemic, the global incidence of AM was estimated to range between 1 and 10 cases per 100,000 individuals [6]. Among patients hospitalized for myocarditis, approximately 30% received a diagnosis of FM, and in pediatric myocarditis hospitalizations, FM accounted for over a third [10]. According to the 2019 Global Burden of Disease study [11], the incidence rate of myocarditis in the 35–39 age group was approximately 6.1 cases per 100,000 men and 4.4 cases per 100,000 women. A similar trend was observed in the 20–40 age group. However, the actual incidence may be underestimated due to the underdiagnosis of certain subacute cases of myocarditis. Viral infections are the most common cause of myocarditis, with Coxsackievirus and Parvovirus B19 (PVB19) considered the most common types of viruses, especially in the United States and Europe [12, 13]. Dengue virus-induced myocarditis has been documented in South Asian countries, such as Pakistan and India [14, 15]. Hepatitis C virus (HCV) is the primary virus responsible for myocarditis in Japan, whereas Chagas disease (CD), caused by Trypanosoma cruzi, is the major cause of myocarditis in Latin America [16]. Different viral infections exhibit seasonal patterns, with enteroviral infections being more prevalent during the summer and fall, while influenza viruses are more prevalent during the winter. Enteroviral myocarditis is more prevalent among young males, and PVB19 and adenoviruses are frequently detected in children with myocarditis [17, 18]. COVID-19 has increased the incidence of myocarditis approximately 15-fold since the beginning of the COVID-19 epidemic [6]. Among COVID-19 hospitalized patients, the incidence of COVID-19 AM is approximately 2.4–4.1 per 1000, of which nearly 40% may be FM [19].

### 1.4 Diagnosis

The symptoms and signs of FM are often atypical and overlap with those of various other cardiac conditions, including acute coronary syndrome (ACS), septic cardiomyopathy, and stress cardiomyopathy, particularly ACS. Consequently, a comprehensive analysis integrating both laboratory tests and imaging studies is required to make the diagnosis [1, 20].

#### 1.4.1 Laboratory Tests

Cardiac injury markers such as creatine kinase (CK), creatine kinase-MB (CK-MB), and cardiac troponin (cTn) are frequently elevated in the early stages of FM and are obtained to make an early diagnosis. B-type natriuretic peptide (BNP) or N-terminal pro-B-type natriuretic peptide (NT-proBNP), peptides synthesized by the heart, serve as potent prognostic indicators for adverse outcomes when serum levels are elevated [21]. These markers signify ventricular dysfunction and myocardial ischemia, providing insight into the extent of myocardial injury. Non-specific inflammation indicators such as C-reactive protein and erythrocyte sedimentation rate may also reflect the level of myocardial inflammation, although normal levels do not necessarily exclude myocarditis [22]. All suspected FM patients should undergo regular monitoring through blood gas analysis, serum lactate (LAC) levels, electrolytes, and liver and kidney functions to evaluate treatment outcomes [1].

#### 1.4.2 Electrocardiography

Electrocardiographic (ECG) abnormalities are observable in up to 85% of AM patients [3]. Among these, ST-segment elevation resembling that of acute myocardial infarction (AMI) is the most prevalent, often involving the inferior and lateral leads [4, 23]. This presents challenges in early diagnosis, necessitating coronary angiography to exclude an AMI. Additional ECG changes that may be present include a QRS width exceeding 120 ms, high-degree or complete atrioventricular block, atrial fibrillation, and ventricular tachycardia/ventricular fibrillation (VT/VF). While the sensitivity of an ECG in diagnosing this condition is relatively high, its specificity is less optimal, necessitating dynamic reassessment to monitor evolving patterns. Arrhythmias are prevalent in FM patients, and the onset of malignant arrhythmias such as complete atrioventricular block, VT/VF often indicates a poor prognosis [24].

#### 1.4.3 Echocardiography

Segmental ventricular wall motion abnormalities, particularly in the inferior and lateral walls, left ventricular wall thickening, and varying degrees of decreased left ventricular ejection fraction (LVEF), are typical echocardiographic features observed in FM patients. Due to its relative accessibility, echocardiography is the preferred initial diagnostic modality for most FM cases. It enables rapid and comprehensive differential diagnoses, encompassing valvular and pericardial diseases, while also assessing cardiac and valvular function and morphology [3]. Echocardiographic changes can also function as prognostic indicators; several studies propose that LVEF can serve as a predictive metric for outcomes in FM patients [3, 25, 26].

#### 1.4.4 Cardiac Magnetic Resonance (CMR)

CMR is a non-invasive, radiation-free technique that offers morphological and functional insights into the patient’s heart, while also detecting myocardial edema, scar formation, or active inflammation. It demonstrates a high diagnostic concordance with pathological biopsy, with an accuracy rate approaching 80% [27]. CMR is valuable for differential diagnosis in clinically suspected FM cases, although its usage is constrained by equipment requirements and time-consuming procedures, limiting its broad application in emergency and clinical settings [7]. When the hemodynamics of FM patients stabilize, CMR assessment can be completed within 2–3 weeks after symptom onset to assess the extent and localization of residual inflammation and myocardial fibrosis [4]. CMR diagnosis primarily adheres to the Lake Louise criteria [28, 29], for diagnosing AM when two or more of the three criteria are met.

#### 1.4.5 Endomyocardial Biopsy (EMB)

EMB is regarded as the gold standard for diagnosing FM [7, 30, 31], offering precise pathological classification to guide targeted treatment. Studies have found that histological subtypes of FM can independently predict prognosis in these patients [32]. For example, patients with giant cell myocarditis exhibit higher early mortality rates or rates of HTx compared to patients with other myocarditis subtypes, emphasizing the need for EMB to definitively identify subtypes. However, the invasive nature of the procedure, coupled with limited sensitivity [31, 33], makes it susceptible to producing false-negative results. Furthermore, it carries an increased potential for complications such as cardiac tamponade and perforation [7, 34], curtailing its widespread application in FM patients.

### 1.5 Prognosis

Although the incidence of FM is relatively low, the early mortality rate can reach as high as 50% [35, 36]. Once patients survive the perilous acute phase, the majority experience favorable long-term outcomes. Studies have indicated that FM patients exhibit better cardiac functional recovery and prognosis compared to NFM patients [35, 37]. McCarthy *et al*. [35] identified 147 patients with AM according to the EMB and the Dallas histopathological criteria, 15 of whom were diagnosed with AFM. 93% of patients with AFM survived successfully without heart transplantation during 11 years of follow-up, compared with 45% of patients with AM. Recent research by Ammirati *et al*. [32] presented divergent findings, noting elevated rates of mortality and requirements for HTx in FM patients in comparison to NFM patients. Upon admission, FM patients exhibited more severe left ventricular dysfunction, although substantial improvement was observed during hospitalization. Nonetheless, in long-term follow-up, the proportion of FM patients with an LVEF below 55% was over three times higher than that of NFM patients (29% vs. 9%). Another retrospective study [10] yielded parallel results; it examined 220 histologically confirmed myocarditis patients presenting with left ventricular dysfunction and found that FM patients had elevated rates of cardiac-related mortality within 60 days post-admission (28.0% vs. 1.8%, *p* < 0.001) and increased 7-year HTx rates (47.7% vs. 10.4%, *p* < 0.001) compared to NFM patients. These prognostic discrepancies may be attributed to varying etiologies. FM often arises from acute triggers such as viral infections, correlating with heightened short-term mortality rates; however, the prognosis significantly improves once the acute etiological factors are mitigated. The manifestation of fulminant symptoms may indicate a more robust immune/inflammatory response in FM patients, suggestive of more efficient viral clearance and is a prognostic marker for eventual myocardial recovery [2]. Variations in histological subtypes also substantially influence the prognosis of FM patients, with multiple studies indicating poorer outcomes for patients with giant cell myocarditis [32, 38, 39, 40].

### 1.6 COVID-19 and Myocarditis

Since the onset of the COVID-19 pandemic caused by severe acute respiratory syndrome coronavirus 2 (SARS-CoV-2), reports of COVID-19 infection and COVID-19 vaccination-associated myocarditis have gradually increased. While COVID-19 primarily affects the respiratory system, it can also impact the cardiovascular system, immune system, and other organ systems. Patients with reported comorbid cardiovascular disease have an increased incidence of COVID-19 and are at risk for a poor prognosis. How, patients without a history of underlying cardiovascular diseases who are affected by COVID-19 may still experience cardiovascular complications such as arrhythmias, myocarditis, and heart failure [41, 42].

COVID-19-associated myocarditis is one of the complications of COVID-19 infection, and the pathogenesis of COVID-19-associated myocarditis is still under investigation. Potential mechanisms currently under consideration include direct invasion of the virus to damage cardiomyocytes, indirect damage due to cellular immune response and cytokine storm resulting from viral infection, and systemic conditions affecting the cardiovascular system, such as severe hypoxia due to viral invasion of other organs [43, 44]. Angiotensin-converting enzyme 2 (ACE2) is a type I transmembrane protein, which is predominantly anchored at the apical surface of the cell. Its major function is converting angiotensin II to angiotensin 1–7 [41]. The ACE2 receptors exhibit high expression levels in the lungs, heart, and blood vessels, and is co-expressed with the serine protease transmembrane protease serine 2 (TMPRSS2) in the lungs (e.g., lung type II alveolar cells, bronchial epithelial cells), heart, intestinal smooth muscle, neurons, and immune cells [41]. This may explain why SARS-CoV-2 is capable of infecting cardiomyocytes and involving multiple organs following COVID-19 infection. SARS-CoV-2 is a new type of RNA virus with an envelope that has protrusions on its surface formed by the outward protrusion of spiny glycoproteins (S proteins). SARS-CoV-2 infects host cells through the binding of its surface S proteins to the ACE2 receptor. The TMPRSS2 serine protease in host cells activates S proteins and cooperates with ACE2 to facilitate cellular invasion by SARS-CoV-2 [45]. The assembly of the virus in the host cell results in the release of the virus, leading to apoptotic lysis and subsequent cardiac antigen release. This can, in turn, elicit the release of inflammatory factors, including interleukins (interleukin-1β (IL-1β), interleukin-6 (IL-6), tumor necrosis factor-α (TNF-α)) [41], ultimately activating T-lymphocyte-mediated cellular immunity. This immune response may further exacerbate myocardial damage. IL-6 is a significant mediator of the cytokine storm [46]. This leads to the activation of T-lymphocytes and the release of cytokines, resulting in a vicious cycle of positive feedback between the immune response and myocardial injury.

Although COVID-19-associated myocarditis is a relatively rare complication of COVID-19, COVID-19 infection complicated by myocarditis increases mortality. A retrospective cohort study in Germany analyzed AM patients hospitalized between 2006–2019 and AM patients hospitalized in 2020 (with or without COVID-19). Compared with the 2006–2019 myocarditis reference cohort, patients with acute myocarditis in 2020 had significantly higher mortality rates regardless of whether they were infected with COVID-19 or not. In-hospital mortality rates for patients with acute myocarditis infected with COVID-19 were more than six times higher than for the non-COVID-19 reference cohort (13.54% vs. 2.21%) [47]. The mortality rates of COVID-19 FM and COVID-19 vaccine-associated FM were reported to be similar (27.7% vs. 27.8%), but patients with COVID-19 FM have more severe disease [48]. The immune response to the SARS-CoV-2 Spike protein may be the pathophysiology underlying COVID-19 FM and COVID-19 vaccine-associated FM [48]. The similar mechanism may account for the similarity in clinical presentation and mortality between the two diseases. The number of studies on the long-term prognosis of COVID-19 infection is still limited. A large cohort study of long-term outcomes of cardiovascular complications after the acute phase of COVID-19 infection confirmed a significantly higher burden of cardiovascular-related complications in survivors at both 30 days and 1 year after infection with COVID-19, despite the absence of prior risk factors or history of cardiovascular disease in these patients, even in those who did not need to be hospitalized after infection with COVID-19 [47, 49]. COVID-19 infection increases the burden of AM and other related cardiovascular diseases. Treatments to reduce the incidence of cardiovascular complications and improve the long-term prognosis of AM patients after COVID-19 are still being explored.

## 2. Treatment and Management of FM

### 2.1 Treatment Strategies for FM

Current treatment strategies for FM primarily center around symptomatic supportive care, encompassing general supportive care, antiviral therapy, immunomodulatory treatments, vasoactive agents, and MCS. However, the exact therapeutic regimen remains uncertain, particularly concerning the application of immunomodulatory treatments. Intravenous immunoglobulin (IVIG) has exhibited anti-inflammatory, immunomodulatory, and antioxidative stress properties that ameliorate myocardial cell injury during the acute phase, contributing to improved left ventricular function and reduced incidence of malignant arrhythmias [50, 51]. Similarly, glucocorticoids (GCs) have shown anti-inflammatory and immunosuppressive effects [1]. Several studies have reported the protective effects of IVIG and/or glucocorticoids in FM patients [52, 53, 54], while an 11-year retrospective study discovered that high-dose use of GCs or IVIG did not notably impact in-hospital or post-discharge outcomes in pediatric myocarditis patients [55]. A multicenter study also indicated that IVIG treatment has not yet conferred significant survival benefits in AM pediatric patients [56]. As viruses primarily infiltrate the myocardium and extensively replicate during the acute phase, early high-dose use of GCs might facilitate viral replication and impede viral clearance. However, they do possess inhibitory effects on the excessive immune response that ensues, thereby safeguarding the heart from auto-immune attacks. Subsequent large-scale, prospective, long-term studies are necessary to clarify the potential survival advantages of immunomodulatory treatments.

According to the Chinese Expert Consensus on the Diagnosis and Treatment of Fulminant Myocarditis [1], comprehensive treatment should commence as early as possible for FM patients, underscoring that life-supporting treatments (circulatory support, respiratory support, and renal replacement therapy) constitute the cornerstone of all therapeutic measures. For FM patients who remain hemodynamically unstable despite maximal medical therapy, MCS is the pivotal treatment. Currently, MCS primarily encompasses intra-aortic balloon pumping (IABP), ECMO, ventricular assist devices (VAD), and Impella support, with ECMO serving as the primary treatment modality for these critically ill patients [57, 58, 59], particularly when hemodynamics are not improved following IABP support [1].

### 2.2 Role and Clinical Efficacy of ECMO

The ECMO system primarily consists of arteriovenous cannulation, connecting tubes, a centrifugal pump, an oxygenator, oxygen supply tubes, and monitoring systems. The fundamental principle involves withdrawing venous blood from the body, passing it through a membrane oxygenator for oxygenation and removal of carbon dioxide, and then reintroducing the oxygenated blood back into the body using a centrifugal pump. This process ensures systemic oxygenation and hemodynamic support. Two main modes of ECMO exist: veno-venous and veno-arterial. FM patients experiencing pump failure typically utilize VA-ECMO for respiratory and circulatory support, affording rest for the failing heart and creating conditions conducive to myocardial recovery. In FM patients with concurrent CS and severe cardiac dysfunction, ECMO can function as a bridge to cardiac transplantation or eventual recovery [60].

As ECMO technology has advanced rapidly and management strategies have evolved, its application in FM has become more widespread. Current research suggests that adult FM patients receiving ECMO exhibit in-hospital survival rates ranging from 55.7% to 75.5% [52, 61, 62, 63], while pediatric FM patients show survival rates of 68.8% to 83.3% [64, 65, 66, 67]. Compared to outcomes in other cardiac conditions treated with ECMO, FM patients demonstrate a more favorable prognosis after ECMO intervention. A meta-analysis conducted by Alba *et al*. [68] indicated that the short-term mortality rate for FM patients was 40% (95% CI 33–46%), which was lower than that for AMI patients (60%; 95% CI 57–64%) and heart failure patients (53%; 95% CI 46–59%). This discrepancy may be attributed to the reversible nature of most FM cases. Timely interventions to maintain hemodynamic stability and organ perfusion are likely to lead to successful myocardial recovery [58], potentially contributing to the lower mortality rate observed in FM patients following VA-ECMO support.

Although ECMO’s role in FM patient care has been documented in several recent studies, the reported survival rates of FM patients receiving ECMO support from different centers vary, indicating a need for further improvement. Early identification of prognostic risk factors associated with FM patients receiving ECMO support and subsequent interventions are pivotal for enhancing outcomes in these high-risk patients. A retrospective analysis by Chong *et al*. [63] involving 35 adult FM patients who underwent VA-ECMO treatment revealed no significant differences between the survival and non-survival groups in terms of age, sex, cardiac rhythm, and hemodynamic status. Both in-hospital survival and 1-year follow-up survival was 57.1%. Elevated peak troponin I (TnI) and 24-hour LAC levels emerged as predictors of in-hospital mortality, suggesting that patients with increased TnI and LAC levels 24 hours post-ECMO support should consider early placement of left ventricular assist devices (LVAD) or immediate HTx. Notably, no patients in this single-center study received either LVAD or urgent HTx.

A study exploring factors related to in-hospital mortality among pediatric FM patients receiving VA-ECMO found that pre-ECMO LAC levels (cutoff value at 79.8 mg/dL) and post-ECMO LVEF (cutoff value at 39%) served as predictive indicators for mortality during hospitalization [31]. Another analysis by Xie *et al*. [25] examined clinical data from 37 children diagnosed with FM to identify independent predictors influencing in-hospital mortality. 25 children in the survivor group were successfully discharged from the hospital after a series of active treatments, including the use of ECMO, high-dose IVIG, GCs, and continuous renal replacement therapy (CRRT). The study found ECG abnormalities such as tachycardia, conduction blocks, and ST-T changes in FM patients. Admission levels of CK and myoglobin (MYO) were significantly higher in the non-survival group than in the survival group, whereas procalcitonin and LVEF levels were notably lower. Multivariate regression analysis highlighted MYO and LVEF as critical predictors of death. The combined diagnosis of MYO and LVEF demonstrated higher predictive value and sensitivity. The study categorized patients based on MYO levels into low-MYO (≤210 µg/L, n = 23) and high-MYO (≤210 µg/L, n = 14) groups, revealing an in-hospital mortality rate of 4.3% for the low-MYO group compared to 78.6% for the high-MYO group after adjusting for age and sex. MYO is a hemoglobin that exists in the cytoplasm of cardiomyocytes and skeletal muscle fibers, whose function is to transport and store oxygen. Elevated early MYO levels signified greater degrees of hypoxia and myocardial injury, emphasizing the need for prompt and effective oxygen supplies and maintenance of vital organ perfusion. In another investigation by Lee *et al*. [69], clinical data from 100 FM patients were retrospectively reviewed to assess patient prognosis and identify risk factors related to in-hospital mortality among those receiving ECMO support; 71 of these patients received ECMO assistance. Patients in the ECMO group exhibited worse myocardial enzyme levels, LAC levels, LVEF, and Sequential Organ Failure Assessment (SOFA) scores than those in the non-ECMO group on admission. In-hospital mortality rates were 28.2% (20/71) and 6.9% (2/29) for the two groups, with an overall mortality rate of 22%. The median follow-up time was 456 days (99–1338 days). No significant difference was observed in the median New York Heart Association (NYHA) class or LVEF among survivors of both groups, suggesting that ECMO may confer survival benefits for FM patients requiring MCS. However, the study did not evaluate other long-term prognostic indicators, and future research is needed to further assess the quality of life and complications in these survivors. The study also identified that SOFA scores (cutoff value at 12) and CK-MB levels (cutoff value at 94.74 ng/mL) significantly correlated with in-hospital mortality, indicating that ECMO support should be considered for FM patients with SOFA scores above 12 and CK-MB levels above 94.74 ng/mL at admission.

Kuo *et al*. [70] analyzed data from 68 adult patients with AFM to investigate risk factors for weaning from ECMO and in-hospital mortality in patients with FM caused by viral infection, 33 of whom were treated with ECMO. Groups were based on whether the etiology was determined to be a viral infection. Eight patients were in the virus group. The results of the study showed an overall survival rate of 54.5%. A confirmed viral etiology, peri-ECMO renal replacement therapy (RRT), positive end-expiratory pressure (PEEP) ≥8 cm $H_{2}$O at 24 h after ECMO therapy were significant predictors of in-hospital mortality, while peri-ECMO RRT was a negative prognostic factor for weaning from ECMO. However, the study was retrospective from a single center with a small sample size, which may have contributed to a selection bias that affected the study’s outcome.

A recent large-scale, multicenter retrospective analysis involving 221 adult FM patients [71] revealed that cardiac arrest prior to ECMO initiation, LAC levels, and arterial blood gas pH values within 24 hours post-ECMO initiation were independent risk factors predicting 90-day mortality. Cardiac arrest prior to ECMO initiation led to a 2.5-fold increased risk of 90-day mortality. Given that survival rates following cardiac arrest due to circulatory failure and severe hypoperfusion can be as low as 13–18% [72, 73], early ECMO initiation is deemed essential. In this study, the 90-day survival rate for FM patients receiving ECMO was 71.9%, aligning with previous reports. However, the study could not evaluate long-term prognosis due to the absence of data on factors potentially related to patient outcomes, such as histological subtypes, timing of ECMO cannulation, blood loss, transfusion volumes, and the incidence of malignant arrhythmias such as VT/VF. The predictors of hospital mortality in FM patients supported with ECMO are summarized in Table 1 (Ref. [31, 63, 69, 70, 71].

**Table 1.** **Overview of studies about the outcomes and predictors of hospital mortality in FM patients supported with ECMO**.

Year	Study design	ECMO/${\mathrm{Total}}^{\left(1\right)}$	Patients type	ECMO weaning, n (%)	ECMO ${\mathrm{Survival}}^{\left(2\right)}$, n (%)	VAD/heart ${\mathrm{transplantation}}^{\left(3\right)}$, n	Survival to ${\mathrm{discharge}}^{\left(4\right)}$, n (%)	Predictors	Reference
2018	Retrospective single-center	35	All adults	N/A	20/35 (57.1)	0	20/35 (57.1)	Post-ECMO peak TnI, Post-ECMO 24 h LAC	[63]
2020	Retrospective cohort	33	All children	N/A	23/33 (69.6)	0	23/33 (69.6)	Pre-ECMO lactate ≥79.8 mg/dL, Post-ECMO LVEF <39%	[31]
2021	Retrospective single-center cohort	71/100	68 adults, 32 pediatrics	N/A	51/71 (71.8)	8 VAD/heart transplantation	78/100 (78)	SOFA scores ≥12 (the worst values within 24 h from ICU admission), CK-MB ≥94.74 ng/mL at ICU admission	[69]
2023	Retrospective	33	All adults	19/33 (57.6)	18/33 (54.5)	2 LVAD+heart transplantation	18/33 (54.5)	confirmed viral etiology, Peri-ECMO RRT, PEEP ≥8 cm $H_{2}$O in the ventilator settings at 24 h after ECMO	[70]
2023	Retrospective multicenter	221	All adults	186/221 (84.2)	159/221 (71.9)	N/A	159/221 (71.9)	Prior ECMO ${\mathrm{CA}}^{\left(5\right)}$, Lactate concentration ≥3.0 mmol/L at 24 h post-ECMO ${\mathrm{initiation}}^{\left(5\right)}$, arterial blood gas pH values <7.35 at 24 h post-ECMO ${\mathrm{initiation}}^{\left(5\right)}$	[71]

Abbreviations: FM, fulminant myocarditis; ECMO, extracorporeal membrane oxygenation; VAD, ventricular assist device; N/A, not applicable; TnI, troponin I; LAC, lactic acid; LVEF, left ventricular ejection fraction; SOFA, Sequential Organ Failure Assessment; ICU, intensive care unit; CK-MB, creatine kinase MB fraction; LVAD, left ventricular assist device; RRT, renal replacement therapy; PEEP, positive end-expiratory pressure; CA, cardiac arrest. ^(1)^ Expressed as fraction of ECMO patients to all patients included in the study. If all patients are ECMO patients, only one number is reported. ^(2)^ Expressed as the fraction of survivors to all ECMO patients included in the study. ^(3)^ Expressed as the fraction of VAD/heart transplantation applied in all ECMO patients in the study. ^(4)^ Expressed as the fraction of survivors to all patients included in the study. ^(5)^ Expressed as the predictor only associated with 90-day survival rate.

### 2.3 Timing for Initiation of ECMO

Currently, there is no established set of guidelines or consensus regarding the ideal timing for initiating VA-ECMO. Different medical centers exhibit varying timing strategies, primarily guided by the patient’s hemodynamic status and individual institutional criteria for instituting ECMO. Premature initiation might lead to unnecessary complications, while delayed initiation could hinder patient recovery. Studies suggests that the principle of “the earlier, the better” holds true for patients with CS [74]. A multicenter study by Lee *et al*. [75] categorized patients into early (<0.9 hours), intermediate (1–2.2 hours), and late (<2.2 hours) initiation groups based on the time from the onset of shock the initiation of ECMO. The results underscore that outcomes are notably better for patients in the early initiation group (0.6 hours) in comparison to those in the intermediate (1.4 hours) and late (5.1 hours) groups with a significant reduction in both the 30-day mortality rate and the all-cause mortality rate at 1 year. The early initiation of ECMO did not increase the rate of complications, such as hemorrhagic or ischemic events.

Early identification of patients with CS and early initiation of ECMO may provide a survival benefit. Pre-ECMO CA has been shown to be an independent predictor of in-hospital mortality in patients with CS [76]. When cardiac output decreases after the onset of CA, the blood supply and circulation to the brain are decreased, resulting in immediate disruption of brain activity, which, if left untreated, can lead to irreversible brain damage or even brain death. The longer the duration of absent perfusion or hypoperfusion after CA, the less likely the recovery of neurologic function. When the time from CA to initiation of ECMO (CA-to-ECMO) exceeds 40 minutes in patients who have experienced an out-of-hospital cardiac arrest (OHCA), the probability of a good neurological prognosis can plummet from more than 30% to about 15% [77]. Several small case studies and a large prospective study [78, 79, 80] have also demonstrated that a long duration of cardiopulmonary resuscitation (CPR) is associated with a reduced chance of survival and neurological recovery. In patients with a sustained return of spontaneous circulation (ROSC) after CA and in patients resuscitated with extracorporeal cardiopulmonary resuscitation (ECPR), CA before ECMO is associated with a significantly increased incidence of death from neurologic causes. Early initiation of ECMO before a patient develops CA is beneficial in reducing mortality in patients at high risk for hemodynamic failure [76]. In patients with witnessed OHCA and those <70 years old with a shockable initial rhythm, initiation of ECMO should be considered as early as possible after 10–20 minutes of unsuccessful cardiopulmonary resuscitation [81]. A retrospective study conducted in Korea [82] emphasized that initiating VA-ECMO in CS patients with a vasoactive-inotropic score (VIS) of ≥32 yielded improved in-hospital outcomes, with no significant variance in the overall incidence of ECMO-related complications between low and high VIS groups, suggesting that the VIS score may be a marker for determining the initiation of hemodynamic support for VA-ECMO [83]. Identifying the optimal timing for ECMO initiation to enhance survival outcomes in FM patients remains an area of increased research.

## 3. ECMO-Related Complications

**Fig. 1.**
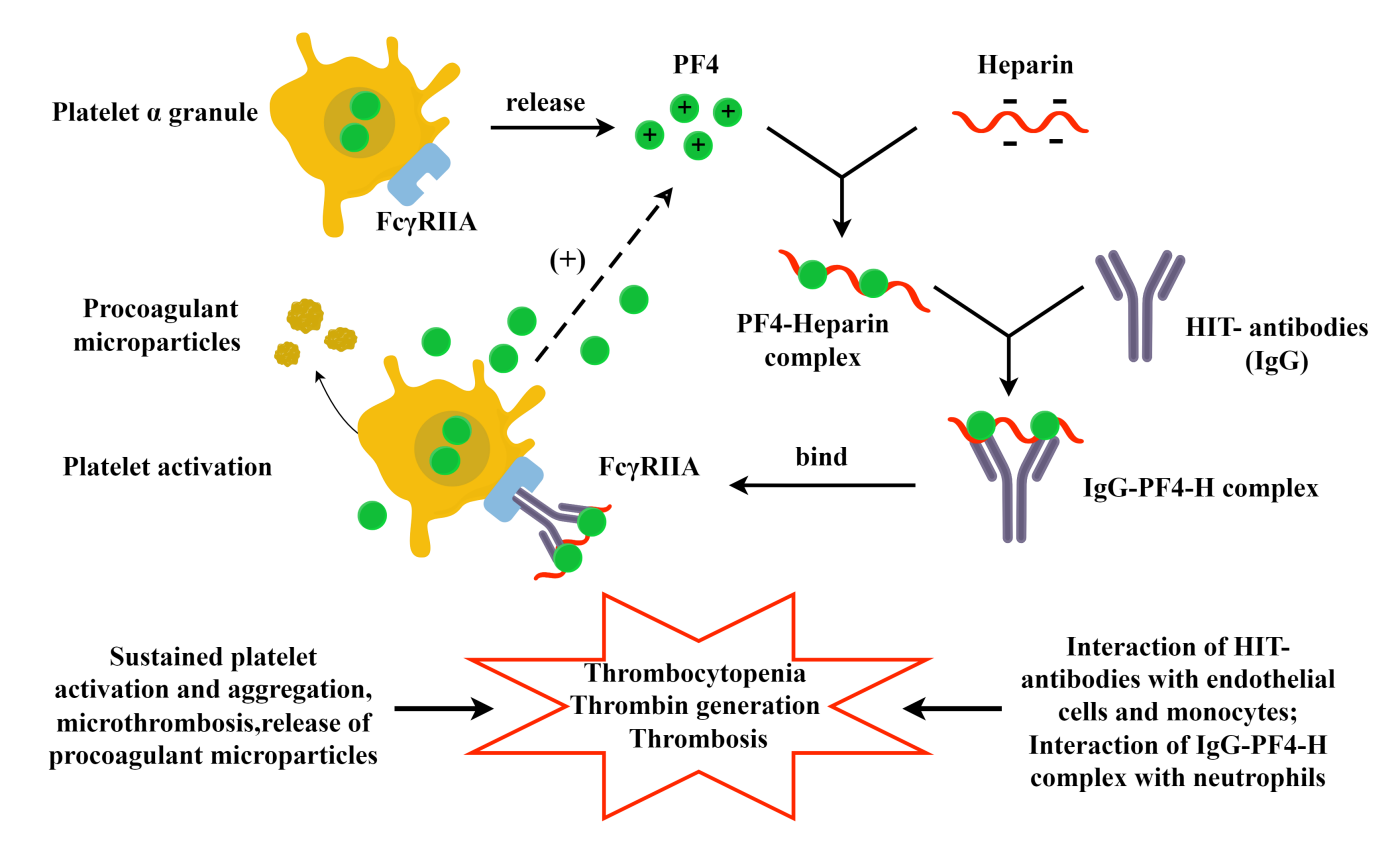
**Pathogenesis of HIT**. PF4 is released from alpha granules in platelets. Positively charged PF4 binds with negatively charged heparin to create the PF4-heparin complex. The IgG HIT antibodies produced bond to this complex to form the IgG-PF4-H complex, which then binds to the platelet Fc receptor. This activates the platelets and leads to the release of procoagulant particles that increase thrombin production. Activated platelets release substantial quantities of PF4, which has a positive feedback effect on HIT. This ultimately results in both thrombocytopenia and thrombosis. The involvement of HIT antibodies with endothelial cells and monocytes, as well as the interaction between IgG-PF4-H complexes and neutrophils, is also implicated in this process. HIT, heparin-induced thrombocytopenia; PF4, platelet factor 4; IgG, immuneglobulin G; IgG-PF4-H complexes, IgG-PF4-Heparin complexes. The figure was drawn by Figdraw.

ECMO provides essential circulatory and respiratory support to patients with FM, yet it is not exempt from inherent complications. Bleeding is one of the most common complications of ECMO, with an incidence ranging from 38–60% [84, 85, 86]. This variation may be due to different approaches to bleeding events and ECMO modalities. The cannulation site is the common source of bleeding [85, 86, 87]. Pulmonary hemorrhage, intracranial hemorrhage, and gastrointestinal hemorrhage are also serious bleeding complications. The process of blood contact with the ECMO circuit causes activation and aggregation of platelets, depletion of coagulation factors, and induces an inflammatory response, resulting in a hypercoagulable state. In order to prevent the occurrence of thromboembolism in the circuit, anticoagulation with heparin or direct thrombin inhibitors (bivalirudin, argatroban, etc.) needs to be initiated during ECMO support. Activated clotting time (ACT) and activated partial thromboplastin time (APTT) are monitored at regular intervals to assist in determining the effect of anticoagulation and adjusting the anticoagulation strategy. Balancing the risk of bleeding and thrombosis is an important issue during ECMO support. Heparin is by far the most common anticoagulant, but heparin-induced thrombocytopenia (HIT) is the most serious complication of heparin anticoagulation. HIT is an antibody-mediated adverse reaction to heparin that occurs during the use of heparin. It is usually characterized by a decrease in platelet count, which can trigger the formation of venous and arterial thrombosis, and can even lead to death. HIT can be mainly categorized into HIT type 1 and HIT type 2. HIT type 1, also known as heparin-associated thrombocytopenia (HAT), is usually mild, transient, and asymptomatic, usually presenting as a mild decrease in platelets that recovers on its own without treatment, and is the most common type of thrombocytopenia. In contrast, HIT type 2 is usually accompanied by significant platelet reduction, and is an immune, antibody-mediated response [88]. Thrombosis and associated embolic complications are the leading cause of death in these patients. The occurrence of HIT type 2 is associated with PF4 autoantibodies after exposure to heparin (Fig. 1). The production of platelet factor 4 (PF4) released from platelet alpha granules binds to heparin to form the PF4-heparin complex, which can stimulate the immune cell response to produce the immuneglobulin G (IgG) HIT antibodies. The Fc fragment of IgG binds to the FcγRIIA receptor on platelets, causing strong platelet activation and aggregation, resulting in thrombocytopenia, increased microparticle production, and escalated thrombin generation. Activated platelets continue to release PF4, which forms more complexes with heparin, activating more platelets and creating a positive feedback loop [89]. Furthermore, HIT antibodies activate endothelial cells and monocytes, resulting in increased thrombin generation and a higher risk of thrombosis in patients with HIT. The HIT immune complex can trigger the activation of neutrophils, promoting thrombosis. The incidence of HIT is approximately 0.2–5%, with a higher incidence in adults than in children [88]. In patients with a high suspicion of HIT, heparin should be discontinued immediately and anticoagulation should be replaced with a direct thrombin inhibitor.

Acute kidney injury (AKI) is one of the common complications in patients receiving ECMO therapy, and it has been reported that the incidence of AKI after receiving ECMO-assisted therapy can be as high as 60, and is associated with a poor prognosis [90, 91]. The occurrence of AKI is associated with ischemia-reperfusion injury, the inflammatory response, hemolysis, and other factors, and the type of ECMO. Some studies have shown that the incidence of AKI is higher in VA-ECMO patients than in VV-ECMO patients [92], which may be due to the fact that the blood flow treated with VA-ECMO comes from retrograde non-pulsatile blood flow provided by the ECMO circuit and mixes with antegrade flow from the heart [93]. The two converge to form the watershed point of blood flow, and the blood flow at the distal end of the watershed comes from the ECMO circuit, so renal perfusion in patients who undergo VA-ECMO is more affected by the non-pulsatile flow provided by ECMO. In contrast, VV-ECMO is usually applied to patients with severe respiratory failure, where the blood flow is mainly pulsatile blood flow from the heart, which has less impact on renal perfusion [94]. Pulsatile blood flow better protects renal perfusion [93]. Continuous renal replacement therapy (CRRT) is an important method for treating ECMO-related AKI. CRRT can reduce the volume load of patients, and removing metabolic wastes and toxins from the body, and at the same time, correct the water-electrolyte disorders, which is conducive to the improvement of renal function. Fluid overload/management, AKI, and correction of electrolyte disturbances are currently the main indications for the application of CRRT in ECMO patients [95]. Common modalities of CRRT include continuous veno-venous hemofiltration (CVVH) and continuous veno-venous hemodialysis filtration (CVVHD) CVVH has been associated with lower mortality in AKI patients treated with ECMO compared to CVVHD [91]. When fluid overload or severe AKI occurs, CRRT therapy should be initiated as early as possible.

One important complication that arises during peripheral VA-ECMO application is left ventricular distention (LVD), with an incidence ranging from 10% to 60% [96]. Due to retrograde aortic flow facilitated by peripheral VA-ECMO, left ventricular afterload is further increased along with wall stress, leading to left ventricular dilatation, elevated left atrial pressure, and pulmonary edema. In severe cases, this can even result in aortic valve closure during systole, left ventricular stasis, and thrombus formation, further worsening ventricular function and hindering myocardial recovery. The outflow cannula for central ECMO is usually inserted in the ascending aorta, which can provide more physiological antegrade blood flow. Therefore, the degree of increase in left ventricular afterload and the rate of related complications may be lower compared to peripheral VA-ECMO [97]. Djordjevic *et al*. [98] showed that central ECMO blood flow is associated with better left ventricular decompression, suggesting that central ECMO may have some left heart decompression effect. However, another study [99] indicates that either peripheral or central cannulation negatively affects left ventricular contraction, and both can lead to some degree of left ventricular distension. Butthese two studies are animal trials, and more studies are needed for further validation. FM patients undergoing central or peripheral VA-ECMO support are prone to varying degrees of LVD. In fact, not all cases require immediate intervention, as approximately 16% necessitate timely management [100]. The decision for left ventricular decompression is contingent upon achieving a balance between the forward flow from the heart pump and the ECMO-supported retrograde flow. Moderate instances of LVD are tolerable, and precise identification of patients who might benefit from ventricular decompression is pivotal. Diagnostic tools such as echocardiography, chest radiographs [100, 101, 102, 103], and chest ultrasound [97, 104] aid in assessing the severity of LVD.

Current approaches for left ventricular decompression include pharmacotherapy (inotropes [81, 97, 100, 105], diuretics, etc.), positive-pressure mechanical ventilation [106], optimizing ECMO flow rates, and percutaneous or surgical decompression techniques (e.g., IABP; Impella; percutaneous atrial septostomy; percutaneous left heart and pulmonary artery drainage; direct surgical superior vena cava to pulmonary artery drainage). Non-invasive strategies are favored, and ECMO parameters should be adjusted to achieve optimal flow rates that ensure systemic perfusion while minimizing detrimental afterload effects. Lower flow rates (<2.2 L/(min⋅$m^{2}$)) have been reported to decrease the occurrence of LVD while maintaining adequate organ perfusion [107]. Percutaneous atrial septostomy is among the initial ventricular decompression methods and has demonstrated efficacy in adults [108, 109] and children [109, 110], particularly in neonates [111]. Data from computer model studies also supports the utility of the percutaneous atrial septostomy [105]. However, it also entails a heightened risk of cardiac perforation, pericardial tamponade, valvular injury, and embolic events, rendering its application a subject of debate [96, 97].

Percutaneous trans-atrial septal left atrial pulmonary artery venting achieves a comparable venting effect on the left ventricle (LV) to atrial septostomy. However, the blood flow drained to the venous side of the ECMO circuit is contingent upon the dimensions of the cannula and tubing. Using a 22 Fr cannula can effectively reduce the left ventricular load, resulting in PCWP reductions ranging from 4–17 mmHg [112, 113]. Transaortic catheter venting (TACV) is one of the methods of left ventricular venting, which can be performed by placing a pigtail catheter (5-7 Fr) into the aorta though femoral artery under esophageal ultrasound or X-ray guidance [114, 115]. However, due to the high risk of hemolysis and the small size of the catheter for percutaneous drainage which limits the maximum volume of drainage, this type of method is not recommended for routine use [116].

Regarding the timing for left ventricular decompression, no universally accepted standard exists. A large international multicenter study indicated that early ventricular decompression (initiated either pre-ECMO or within 2 hours post-VA-ECMO initiation) is linked to lower 30-day mortality rates in patients with CS [117]. Conversely, no such benefit was observed in groups with delayed decompression (initiated 2 hours post-VA-ECMO). Al-Fares *et al*. [118] found that decompression performed either pre-ECMO or within 12 hours post-VA-ECMO initiation led to improved weaning rates and short-term mortality in CS patients, but this advantage was not evident in myocarditis patients. Subsequent research is vital to determine the optimal timing for left ventricular decompression in FM patients and to develop best-practice protocols.

## 4. ECMO and Other MCS Devices

### 4.1 Intra-Aortic Balloon Pumping (IABP)

The IABP plays a pivotal role as a temporary MCS technology, initially demonstrating success in rescuing patients with CS [119]. The mechanism of the IABP involves rapid inflation of the balloon during diastolic, leading to an elevation in aortic diastolic pressure which augments coronary perfusion and contributes to improved myocardial oxygenation. During systole, the balloon rapidly deflates, resulting in a reduction in aortic pressure. This action alleviates left ventricular afterload, subsequently reducing cardiac workload and myocardial oxygen consumption. In patients with FM complicated by CS, IABP provides circulatory support, minimizing the necessity for vasoactive medications and assisting patients during the acute phase [1]. The statement *Recognition and Initial Management of Fulminant Myocarditis* published by The American Heart Association (AHA) summarizes the general approach to the initial support of patients in cardiogenic shock. The IABP used for temporary mechanical circulatory support, is among the recommended management strategies [2].

Previously, the IABP was recommended as a first-tier treatment for CS in both the US and European guidelines [120, 121]. However, recent results from the IABP SHOCK II clinical trials [122, 123, 124] have raised doubts about its efficacy in patients with AMI-CS. The IABP SHOCK II trial demonstrated that the use of IABP did not have a significant impact on reducing mortality rates at 30-day, 1-year, and 6-year intervals in patients with AMI-CS. IABP did not significantly improve 5-year survival rates or decrease the incidence of major adverse cardiac and cerebrovascular event (MACCE) in the IMPRESS randomized trial comprising patients who developed severe CS after AMI [125]. The findings of these studies are quite different from those of previous studies, which may be related to the timing of the IABP intervention [117, 126]. Patients in the IABP-SHOCK II trial who were in the IABP group might have needed vasoactive medications to sustain hemodynamics before undergoing percutaneous transluminal coronary intervention (PCI) or coronary artery bypass grafting (CABG). The potential unfavorable effects of using vasoactive medications may have outweighed the potential benefits of the IABP [127]. Furthermore, if CS patients in the IABP group required IABP implantation due to the deterioration of their condition during the procedure, the optimal timing of IABP placement might have also been affected [128]. In addition, patients in IABP-SHOCK II were not risk-stratified, and therefore patients who would benefit most from IABP use were not clearly identified. A retrospective analysis [129] investigated the correlation between IABP application and mortality for patients with AMI-CS categorized by the Society for Cardiovascular Angiography and Interventions (SCAI). The results indicated that the IABP was linked to decreased mortality for patients with stage A/B shock while excluding those with stage C/D/E. Therefore, early identification of patients who may benefit from IABP application could potentially enhance CS patient outcomes.

The integration of IABP with VA-ECMO can attenuate the increase in left ventricular afterload caused by VA-ECMO by decreasing systemic afterload. The IABP can provide pulsatile blood flow during VA-ECMO support, which facilitates improved organ perfusion [130]. In addition, it also can prevent the development of hydrostatic pulmonary edema [131]. Whether the use of VA-ECMO in combination with IABP can reduce mortality and improve prognosis in patients with CS is still under investigation. A meta-analysis by Zeng *et al. * [132] examined whether combining ECMO with IABP improves outcomes in CS in comparison to ECMO alone. The findings indicated that the simultaneous application of ECMO and IABP could more effectively enhance in-hospital survival rates among CS patients. However, this study did not specify the sequential order of device placement for IABP and ECMO, and the patients exhibited considerable heterogeneity in terms of the underlying causes and severity of CS, potentially affecting the reliability of the results. Conversely, a study by Lin *et al*. [133] suggested that the combined use of IABP and ECMO did not significantly improve survival rates for patients with circulatory failure. Their retrospective analysis encompassed clinical data from 529 CS patients—227 treated with ECMO and 302 treated with a combination of IABP and ECMO. The results indicated no substantial differences between the two groups in terms of two-week all-cause mortality, the incidence of multi-organ failure, or other complications. The study also suggested that co-administration of IABP did not significantly decrease LAC levels, implying limited effectiveness in enhancing microcirculation and tissue perfusion to prevent organ failure. Similarly, Wang *et al*. [134] conducted a meta-analysis involving 12 observational studies encompassing 3704 patients to assess the efficacy of the IABP combined with VA-ECMO versus VA-ECMO alone in treating patients with CS or cardiac arrest. Their findings demonstrated that the mortality rate in the combined IABP and VA-ECMO group was 59.7%, compared to 65.8% in the VA-ECMO group. Moreover, the success rate for weaning off VA-ECMO was significantly higher in the combined treatment group (77.9% vs. 61.2%; *p* < 0.001). While the combination of IABP and VA-ECMO appears to enhance the success rate of weaning off VA-ECMO, it does not substantially improve in-hospital mortality rates for patients with CS or cardiac arrest. The benefit of IABP in saving patients with CS remains controversial. Recently, a Japanese retrospective cohort study [135] identified 1650 CS patients to investigate the effect of ECMO combined with IABP on mortality in CS patients and created 533 pairs based on propensity score matching. The results of the propensity score matching analysis found that all-cause 28-day mortality and in-hospital mortality were significantly lower in the ECMO+IABP group than in the ECMO alone group. This finding was also confirmed by the COX regression analysis. In addition, the weaning rate in CS patients was higher in the ECMO+IABP group. The benefit of ECMO+IABP over ECMO alone in reducing mortality in patients with CS was also supported in a meta-analysis by Russo *et al*. [136].

Although the use of IABP in patients with CS remains controversial, it continues to be one of the most extensively utilized mechanical assist devices in clinical practice. Nonetheless, a recent study [137] indicates that IABP may provide some protective benefits for patients with myocarditis. However, there is a lack of large-scale randomized controlled trials in patients with FM-combined CS to determine the effectiveness of the IABP. Thus, further studies are needed to clarify the efficacy of the IABP in these patients.

### 4.2 Impella

VAD represent a subset of MCS systems designed to partially or completely replace cardiac function. Impella, a micro axial left ventricular-aortic pump, offers hemodynamic support similar to conventional VADs but distinguishes itself through its compact size and minimally invasive nature. The device functions by drawing blood from the left ventricle via a catheter and then pumping it directly into the aorta at elevated flow rates (with a maximal output ranging from 2.5 to 6.2 L/min) [138]. This dual action enhances cardiac output while simultaneously reducing left ventricular afterload and lowering myocardial oxygen consumption. In patients with myocarditis who have undergone ECMO treatment, an increase in left ventricular afterload may trigger the onset of an inflammatory response and promote detrimental myocardial remodeling. However, Impella, apart from providing circulatory support, mitigates the afterload, thereby reducing the inflammatory response, which enables the recovery of the myocardium [139, 140]. Annamalai *et al*. [141] studied 34 FM patients with CS who received Impella support and the overall survival rate was 62% (21/34), which is comparable to previously reported survival rates with ECMO therapy alone, as well as a significant improvement in LVEF at discharge in this group of patients. However, the incidence of anemia requiring transfusion was nearly 20%, which may be related to Impella-induced hemolysis. Studies indicate that the combined use of VA-ECMO and Impella, referred to as ECpella, might lead to decreased mortality rates in patients with CS [117, 142, 143]. Nevertheless, introducing a second device increases the potential for complications, including hemorrhage, vascular issues, and renal dysfunction. A multicenter retrospective cohort study conducted by Pappalardo *et al*. [143] found that ECpella substantially reduced in-hospital mortality rates (47% vs. 80%, *p* < 0.001) and increased successful bridging to recovery or advanced therapies (such as left ventricular assist device implantation or HTx) at 68% vs. 28% (*p* < 0.001). These advantages are attributed to the Impella ability to mitigate left ventricular afterload associated with VA-ECMO and its consequent complications. However, it is important to note that ECpella might prolong the duration of mechanical ventilation and MCS support, elevate the need for CVVH, and raise the risk of hemolysis. In addition, Impella is expensive, which limits its widespread clinical use. Current research on the use of ECpella in the treatment of FM consists mainly of case reports [144, 145, 146]. The effectiveness of ECpella requires validation from future prospective randomized studies, which can refine management strategies for FM cases complicated by CS.

## 5. Conclusions

FM is a rare, yet severe clinical syndrome that can lead to adverse outcomes. For patients with FM who have failed conventional treatment, ECMO can provide respiratory and circulatory support, and is a suitable treatment for both adults and children. ECMO is an important means of treating FM, but it isn’t without its challenges, and also is accompanied by some inherent complications, which will require further research to improve patient outcomes. Early identification of FM patients, determining the optimal timing for initiating ECMO, careful management of ECMO procedures, and preventing complications such as LVD are critical factors in improving survival rates. Future research will focus on identifying and validating associated risk factors to further enhance the overall prognosis and clinical outcomes and reduce mortality rates for individuals with FM.

## Abbreviations

FM, fulminant myocarditis; NFM, non-fulminant myocarditis; CS, cardiogenic shock; MCS, mechanical circulatory support; ECMO, extracorporeal membrane oxygenation; AM, acute myocarditis; ICIs, immune checkpoint inhibitors; COVID-19, corona virus disease 2019; PVB19, Parvovirus B19; HCV, Hepatitis C virus; HTx, heart transplantation; ACS, acute coronary syndrome; CK, creatine kinase; CK-MB, creatine kinase-MB; BNP, B-type natriuretic peptide; NT-proBNP, N-terminal pro-B-type natriuretic peptide; LAC, lactate; ECG, electrocardiographic; AMI, acute myocardial infarction; VT, ventricular tachycardia; VF, ventricular fibrillation; RRT, renal replacement therapy; PEEP, positive end-expiratory pressure; LVEF, left ventricular ejection fraction; CMR, cardiac magnetic resonance; EMB, endomyocardial biopsy; SARS-CoV-2, Severe acute respiratory syndrome coronavirus 2; ACE2, angiotensin converting enzyme 2; TMPRSS2, transmembrane protease serine 2; IL-1β, interleukin-1β; IL-6, interleukin-6; TNF-α, tumor necrosis factor-α; IVIG, intravenous immunoglobulin; IABP, intra-aortic balloon pumping; VAD, ventricular assist devices; TnI, troponin I; LVAD, left ventricular assist devices; MYO, myoglobin; SOFA, Sequential Organ Failure Assessment; NYHA, New York Heart Association; CA, cardiac arrest; OHCA, out-of-hospital cardiac arrest; CPR, cardiopulmonary resuscitation; ROSC, return of spontaneous circulation; ECPR, extracorporeal cardiopulmonary resuscitation; VIS, vasoactive-inotropic score; ACT, activated clotting time; APTT, activated partial thromboplastin time; HIT, heparin-induced thrombocytopenia; HAT, heparin-associated thrombocytopenia; PF4, platelet factor 4; IgG, immuneglobulin G; AKI, acute kidney injury; CRRT, continuous renal replacement therapy; CVVH, continuous veno-venous hemofiltration; CVVHD, continuous veno-venous hemodiafiltration; LVD, left ventricular distention; PCWP, pulmonary capillary wedge pressure; TACV, Transaortic catheter venting; MACCE, major adverse cardiac and cerebrovascular event; PCI, percutaneous transluminal coronary intervention; CABG, coronary artery bypass grafting.
